# The Effects of Potassium Cyanide on the Functional Recovery of Isolated Rat Hearts after Ischemia and Reperfusion: The Role of Oxidative Stress

**DOI:** 10.1155/2018/5979721

**Published:** 2018-07-19

**Authors:** Anica M. Petkovic, Vladimir Lj. Jakovljevic, Jovana V. Bradic, Jovana N. Jeremic, Nevena S. Jeremic, Tamara R. Nikolic Turnic, Nemanja U. Jovicic, Vesna Z. Rosic, Ivan M. Srejovic, Vladimir I. Zivkovic

**Affiliations:** ^1^Department of Pharmacy, Faculty of Medical Sciences, University of Kragujevac, Svetozara Markovica 69, 34 000 Kragujevac, Serbia; ^2^Department of Physiology, Faculty of Medical Sciences, University of Kragujevac, Svetozara Markovica 69, 34 000 Kragujevac, Serbia; ^3^Department of Human Pathology, I.M. Sechenov First Moscow State Medical University, Trubetskaya Street 8, Moscow 119991, Russia; ^4^Department of Histology and Embryology, Faculty of Medical Sciences, University of Kragujevac, Svetozara Markovica 69, 34 000 Kragujevac, Serbia

## Abstract

This investigation is aimed at examining the effects of pharmacological PostC with potassium cyanide (KCN) on functional recovery, gene expression, cytochrome c expression, and redox status of isolated rat hearts. Rats were divided into the control and KCN groups. The hearts of male Wistar albino rats were retrogradely perfused according to the *Langendorff* technique at a constant perfusion pressure of 70 cmH_2_O. After stabilisation, control hearts were subjected to global ischemia (5 minutes), followed by reperfusion (5 minutes), while experimental hearts underwent global ischemia (5 minutes) followed by 5 minutes of reperfusion with 10 *μ*mol/L KCN. The following parameters of heart function were measured: maximum and minimum rates of pressure development, systolic and diastolic left ventricular pressure, heart rate, and coronary flow. Levels of superoxide anion radical, hydrogen peroxide, nitrites, and index of lipid peroxidation (measured as thiobarbituric acid-reactive substances) were measured in coronary venous effluent, and activity of catalase was determined in heart tissue. Expression of Bax, Bcl-2, SOD-1, SOD-2, and cytochrome c was studied as well. It was shown that expression of Bax, Bcl-2, and SOD-2 genes did not significantly differ between groups, while expression of SOD-1 gene and cytochrome c was lower in the KCN group. Our results demonstrated that KCN improved the recovery of myocardial contractility and systolic and diastolic function, enhanced catalase activity, and diminished generation of prooxidants. However, all possible mechanisms and potential adverse effects of KCN should be further examined in the future.

## 1. Introduction

The leading causes of morbidity and mortality nowadays are cardiovascular diseases. Acute myocardial infarction probably represents the most often variety within this pathophysiological entity due to reduction of coronary blood flow and myocardial ischemia. Reperfusion strategies can be used for rescuing the myocardium, but immediate reperfusion of ischemic myocardial tissue can paradoxically induce further damage known as the phenomenon of “reperfusion injury.” A lot of effort has been invested to find effective methods to reduce reperfusion injury [1, 2].

Postconditioning (PostC) is a strategy which provides cardioprotection through exposure to sublethal stimulus initiated at the very onset of reperfusion. Two elementary techniques of myocardial PostC involve ischemic PostC and pharmacological PostC [3]. Groundbreaking research which gave the first data about the ischemic PostC was conducted by Zhao and coworkers approximately a decade ago. It was reported that brief episodes of I/R applied at early reflow can be applicable in occasions such as artery bypass surgery, transplantation of organs, and peripheral revascularization [4]. The beneficial effect of ischemic PostC involves postponed reversal of acidosis, activation of protein kinase C (PKC), formation of autacoids, such as adenosine and bradykinin, and release of endogenous opioids [5]. Furthermore, activation of mitochondrial K_ATP_ channel and closure of mitochondrial permeability transition pore may also contribute to cardioprotection [6]. Positive effects of ischemic PostC may be mediated via decreased NO-peroxynitrite signaling, as well as endogenous H_2_S production which stimulates PKC-*α* and PKC-*ε* [7]. In addition, ischemic PostC may diminish the harmful effects of reactive oxygen species (ROS), bearing in mind that oxidative burst during reperfusion remains one of the major factors responsible for reperfusion injury [8]. Ischemic PostC is an adaptive response induced by short periods of ischemia alternating with short periods of reperfusion applied at onset of reperfusion after continuous ischemia; however, the similar effects can be caused by pharmacological agents as well [9].

Cyanide is a rapidly acting as deadly poison that can exist in various forms (gas, solid, and liquid). The main mechanism of its toxicity lies in inhibition of mitochondrial cytochrome c oxidase (CcO), an enzyme involved in formation of terminal complex in the respiratory chain that is integral to the production of adenosine triphosphate (ATP). Consequently, cells are unable to use oxygen and ATP which results in cellular dysfunction and death [10, 11]. Interestingly, potassium cyanide (KCN) applied in a small concentration may attenuate myocardial dysfunction and decrease production of reactive oxygen species (ROS) in a model of heart preconditioning [12].

Regarding all above presented statements, the aim of this study was to examine the effects of KCN in a pharmacological model of PostC on the functional recovery and oxidative stress parameters of isolated rat hearts.

## 2. Materials and Methods

### 2.1. Ethical Approval

The study was performed in the cardiovascular laboratory of the Faculty of Medical Sciences, University of Kragujevac, Serbia. The experimental protocol was approved by the Ethics Committee for the welfare of experimental animals of the Faculty of Medical Sciences, University of Kragujevac. All experiments were performed according to EU Directive for welfare of laboratory animals (86/609/EEC) and principles of Good Laboratory Practice (GLP).

### 2.2. Animals

Sixteen male Wistar albino rats (eight weeks old, body weight 200–250 g, obtained from Military Medical Academy, Belgrade, Serbia) were subjected to the study's protocol. Rats were housed with a temperature adjusted to 22 ± 2°C with 12 : 12 light/dark cycle. They consumed commercial rat food (20% protein rat food, Veterinary Institute Subotica, Serbia) and tap water ad libitum.

### 2.3. Preparation of Isolated Rat Hearts

The hearts of male Wistar albino rats, divided into the control and KCN group (8 in each group), were excised and retrogradely perfused according to *Langendorff* technique (Experimetria Ltd.,1062 Budapest, Hungary). After a short-term narcosis induced by intraperitoneal application of ketamine (10 mg/kg) and xylazine (5 mg/kg) and premedication with heparin as an anticoagulant, animals were sacrificed by cervical dislocation (schedule 1 of the Animals (Scientific Procedures) Act 1986, UK). After urgent thoracotomy and rapid cardiac arrest by superfusion with ice-cold isotonic saline, the hearts were rapidly excised; the aortas were cannulated and retrogradely perfused under a constant perfusion pressure (CPP) of 70 cmH_2_O. The composition of the nonrecirculating Krebs–Henseleit perfusate was as follows (mmol/L): NaCl 118, KCl 4.7, CaCl_2_ × 2H_2_O 2.5, MgSO_4_ × 7H_2_O 1.7, NaHCO_3_ 25, KH_2_PO_4_ 1.2, glucose 11, and pyruvate 2, equilibrated with 95% O_2_ plus 5% CO_2_ and warmed to 37°C (pH 7.4).

### 2.4. Physiological Assay and Experimental Protocol

All study groups underwent 30 minutes of perfusion at a CPP of 70 cmH_2_O (period of stabilisation). In the control group, after the stabilisation period, the hearts were subjected to global ischemia (perfusion was totally stopped) for 5 minutes, followed by 5 minutes of reperfusion. In the KCN group, after the stabilisation period, the hearts underwent global ischemia lasting for 5 minutes and then submitted to 5 minutes of reperfusion with 10 *μ*mol/L KCN [13].

After placing the sensor (transducer BS473-0184, Experimetria Ltd., Budapest, Hungary) in the left ventricle, the following parameters of myocardial function have been measured during stabilisation and during reperfusion: maximum rate of pressure development in the left ventricle (dp/dt max), minimum rate of pressure development in the left ventricle (dp/dt min), systolic left ventricular pressure (SLVP), diastolic left ventricular pressure (DLVP), and heart rate (HR). Coronary flow (CF) was measured flowmetrically in specific points of interest in the control group—at the end of stabilisation, in the first minute of reperfusion (R1), and in the last minute of reperfusion (R2)—and in the KCN group—at the end of stabilisation, in the first minute of reperfusion (R1), and in the last minute of reperfusion (R2). The average heart weight in the control group and the KCN group was very similar (control group—1.1 ± 0.02, KCN group—1.06 ± 0.09).

The following oxidative stress parameters were determined spectrophotometrically (Shimadzu UV-1800, Japan) using collected samples of the coronary venous effluent: the index of lipid peroxidation, measured as thiobarbituric acid-reactive substances (TBARS), nitrite (NO_2_^−^), levels of superoxide anion radical (O_2_^−^), and hydrogen peroxide (H_2_O_2_).

### 2.5. Determination of the Index of Lipid Peroxidation Measured as TBARS

The degree of lipid peroxidation in the coronary venous effluent was estimated by measuring TBARS, using 1% thiobarbituric acid in 0.05 NaOH, which was incubated with the coronary effluent at 100°C for 15 min and measured at 530 nm. Krebs–Henseleit solution was used as a blank probe [14].

### 2.6. Nitrite Determination (NO_2_^−^)

Nitric oxide (NO) decomposes rapidly to form stable nitrite/nitrate products. The NO_2_^−^ level was measured and used as an index of NO production, using Griess's reagent. A total of 0.5 mL of perfusate was precipitated with 200 *μ*L of 30% sulphosalicylic acid, vortexed for 30 min and centrifuged at 3000 × g. Equal volumes of the supernatant and Griess's reagent (containing 1% sulphanilamide in 5% phosphoric acid/0.1% naphthalene ethylenediamine-dihydrochloride) were added, incubated for 10 min in the dark, and measured at 543 nm. NO_2_^−^ levels were calculated using sodium nitrite as the standard [15].

### 2.7. Superoxide Anion Radical Determination (O_2_^−^)

O_2_^−^ levels were measured at 530 nm via a nitro blue tetrazolium reaction in TRIS buffer with coronary venous effluent. Krebs–Henseleit solution was used as a blank probe [15].

### 2.8. Hydrogen Peroxide Determination (H_2_O_2_)

The measurement of H_2_O_2_ was based on the oxidation of phenol red by H_2_O_2_ in a reaction catalyzed by horseradish peroxidase [16]. Two hundred microlitres of perfusate was precipitated using 800 mL of freshly prepared phenol red solution; 10 *μ*L of (1 : 20) horseradish peroxidase (made immediately before use) was subsequently added. For the blank probe, an adequate volume of Krebs–Henseleit solution was used instead of coronary venous effluent. The level of H_2_O_2_ was measured at 610 nm.

### 2.9. Catalase Activity Determination

After accomplishing the experiments, the hearts from all animals were frozen at −80°C, and then a 0.5 section of each tissue was homogenized in 5 mL phosphate buffer pH 7.4 using an electrical homogenizer, on ice. Afterwards, tissue homogenates were centrifuged at 1200 × g for 20 min at 4°C. The resulting supernatants were isolated and stored at −80°C until determination of catalase (CAT) activity. CAT activity was determined according to Aebi. Diluted homogenate of heart tissue (1 : 7 *v*/*v*) was treated with chloroform ethanol (0.6 : 1 *v*/*v*). CAT buffer, prepared samples, and 10 mM H_2_O_2_ were used for determination. Detection was performed at 360 nm. The amount of CAT was expressed as U/g tissue [17].

### 2.10. Expression of Genes in the Heart Tissue

TRIzol reagent (Invitrogen, Carlsbad, CA) according to the manufacturer's instructions was used for isolation of total RNA. Total RNA (*μ*g) was reverse transcribed using High Capacity cDNA Reverse Transcription Kit (Applied Biosystems, Foster City, California, USA). Real-time quantitative PCR was performed using Thermo Scientific Luminaris Color HiGreen qPCR Master Mix (Applied Biosystems, Foster City, California, USA) and mRNA-specific primers for SOD-1, SOD-2, Bax, Bcl-2, and *β*-actin as a housekeeping genes (Invitrogen, Carlsbad, CA) (Table 1). PCR reactions were done in a Mastercycler ep realplex (Eppendorf, Hamburg, Germany). Data were analyzed and relative gene expression was calculated according to Schmittgen and Livak [18].

### 2.11. Immunohistochemistry

After sacrificing, the hearts were extracted, fixated in 10% formaldehyde, and immersed in paraffin. Formalin-fixed paraffin-embedded (FFPE) sections, 5 *μ*m thick, were deparaffinized, rehydrated, and treated with citrate buffer (pH 6.0) in a microwave for antigen restoration. Endogenous peroxidase activity was blocked using hydrogen peroxide (3%). Immunohistochemical staining was performed by incubating FFPE tissue sections with primary mouse anti-cytochrome C antibody (338500, Invitrogen, Carlsbad, CA) overnight at room temperature. Staining was visualized by using Expose Mouse and Rabbit Specific HRP/DAB Detection IHC Kit (ab80436, Abcam, Cambridge, UK). Sections were counterstained with Mayer's hematoxylin and photomicrographed by light microscope (Olympus BX51, Japan) equipped with a digital camera. Results are presented as a mean count of positive stained cells per field at ×40 magnification [19].

### 2.12. Drugs

All drugs were purchased from Sigma–Aldrich Chemie GmbH, Germany.

### 2.13. Statistical Analysis

IBM SPSS Statistics 20.0 for Windows was used for statistical analysis. Three specific points of interest were statistically analyzed in both groups: the first point was stabilisation (S), the second was the first (R1) and the last points of 5-minute reperfusion period (R2). Descriptive statistics were used to calculate arithmetic mean with dispersion measures (standard deviation (SD) and standard error (SE)). Values were expressed as mean ± standard error (SE). Distribution of data was checked by Shapiro–Wilk test. Data was analyzed using a one-way analysis of variance (ANOVA) and the post hoc Bonferroni test for multiple comparisons. Values of *p* < 0.05 were considered to be statistically significant, while values *p* < 0.01 were considered to be highly statistically significant.

## 3. Results

### 3.1. Cardiodynamic Parameters

#### 3.1.1. Maximum Rate of Left Ventricular Pressure Development (dp/dt max)

**Figure 1:**
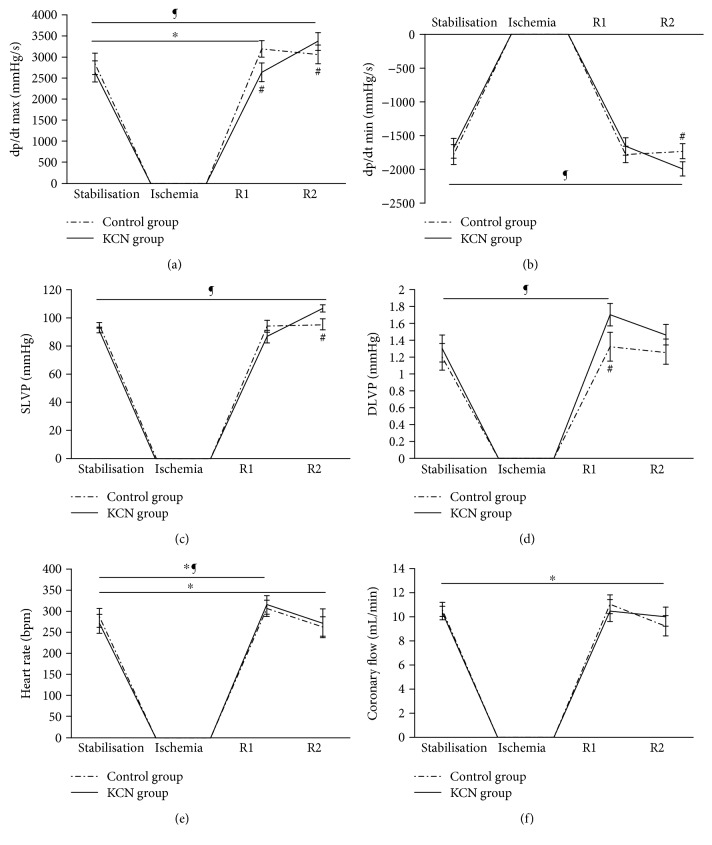
Effects of KCN postconditioning on cardiodynamic parameters. (a) Comparison within and between groups in the value of dp/dt max, (b) comparison within and between groups in the value of dp/dt min, (c) comparison within and between groups in the value of SLVP, (d) comparison within and between groups in the value of DLVP, (e) comparison within and between groups in the value of heart rate, and (f) comparison within and between groups in the value of coronary flow. ^∗^Statistical significance at the level of *p* < 0.05 within the control group; ^¶^statistical significance at the level of *p* < 0.05 within the KCN group; ^#^statistical significance at the level of *p* < 0.05 between the control and KCN groups; data are presented as means ± SE. R1: first minute of reperfusion; R2: last minute of reperfusion.

In the control group, parameter dp/dt max was significantly higher at R1 (3192.14 ± 192.74) compared to S (2839.08 ± 250.01). On the other hand, in the KCN group, dp/dt max values at S (2657.08 ± 252.2) and R1 (2645.56 ± 220.2) were similar; however, a statistically significant increase was noticed at R2 (3370.32 ± 210.3) compared to S (2657.08 ± 252.2). Additionally, when the control and KCN groups were compared, significantly lower values were noticed in the KCN group at R1 (2645.56 ± 220.2 for KCN versus 3192.14 ± 192.74 for control), while values at R2 were statistically higher in the KCN group (3370.32 ± 210.3 for KCN versus 3063.66 ± 225 for control) (Figure 1(a)).

#### 3.1.2. Minimum Rate of Left Ventricular Pressure Development (dp/dt min)

Values of dp/dt min parameter did not vary significantly within the control group (−1780.6 ± 145.96 at S versus −1774.88 ± 122 at R1 versus −1730.98 ± 112.70 at R2). On the other hand, within the KCN group, dp/dt min reached more negative values at R2 (−1989.28 ± 103.1) compared to S (−1690.6 ± 145.96). More negative values of the observed parameter were noticed in the KCN group (−1989.28 ± 103.1) in comparison to the control group (−1730.98 ± 112.70) at R2 (Figure 1(b)).

#### 3.1.3. Systolic Left Ventricle Pressure (SLVP)

After KCN administration, systolic pressure significantly increased at R2 (106.22 ± 2.24) compared to S (90.79 ± 1.68). Values of SLVP did not change significantly in the control group (94.79 ± 1.68 at S versus 93.92 ± 4.24 at R1 versus 95.1 ± 3.85 at R2), as well as between S (90.79 ± 1.68) and R1 (86.48 ± 4.37) in the KCN group. Comparing R2 between the KCN (106.22 ± 2.24) and control groups (95.1 ± 3.85), significantly higher values of SLVP were noticed in the KCN-treated group (Figure 1(c)).

#### 3.1.4. Diastolic Left Ventricular Pressure (DLVP)

DLVP remained unchanged within observed points of interest in the control group (1.2 ± 0.16 at S versus 1.32 ± 0.17 at R1 versus 1.26 ± 0.15 at R2). Values of diastolic pressure significantly increased at R1 (1.7 ± 0.13) in the KCN group compared to S (1.3 ± 0.16). Significant increase of DLVP was noticed in the KCN group at R1 (1.7 ± 0.13) compared to the control group (1.32 ± 0.17) (Figure 1(d)).

#### 3.1.5. Heart Rate (HR)

In the control group, HR significantly increased at R1 (307.28 ± 19.46) and then decreased at R2 (264.4 ± 22.91) compared to S (284.7 ± 22.44). In the KCN group, HR was significantly higher at R1 (314.98 ± 21.75) compared to S (270.7 ± 22.4), while there was no difference between R2 (272.24 ± 33.61) and S (Figure 1(e)).

#### 3.1.6. Coronary Flow (CF)

A significant drop of CF was observed at R2 (9.2 ± 0.88) compared to S (10.6 ± 0.56) in the control group. On the other hand, in the KCN group, CF remained constant during the experiment (10.3 ± 0.56 at S versus 10.52 ± 0.91 at R1 versus 10 ± 0.79 at R2) (Figure 1(f)).

### 3.2. Oxidative Stress Parameters

#### 3.2.1. Levels of Index of Lipid Peroxidation (Measured as Thiobarbituric Acid-Reactive Substances (TBARS))

**Figure 2:**
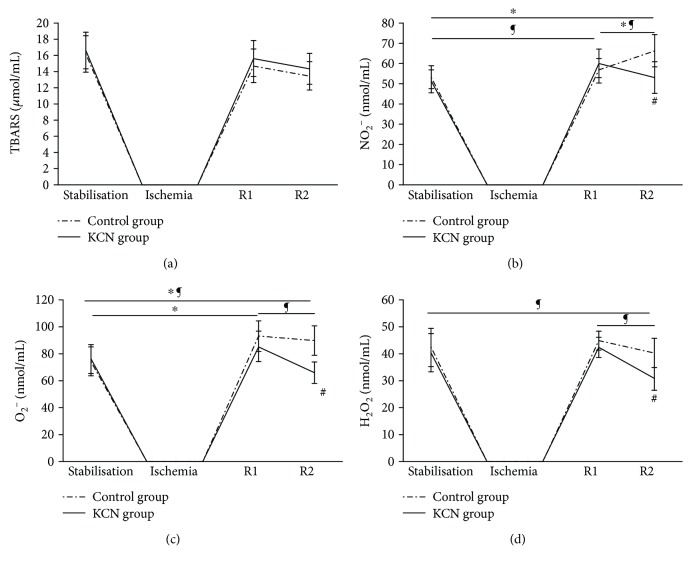
Effects of KCN postconditioning on oxidative stress parameters. (a) Comparison within and between groups in the value of TBARS, (b) comparison within and between groups in the value of NO_2_^−^, (c) comparison within and between groups in the value of O_2_^−^, and (d) comparison within and between groups in the value of H_2_O_2_. ^∗^Statistical significance at the level of *p* < 0.05 within the control group; ^¶^statistical significance at the level of *p* < 0.05 within the KCN group; ^#^statistical significance at the level of *p* < 0.05 between the control and KCN groups; data are presented as means ± SE. R1: first minute of reperfusion; R2: last minute of reperfusion.

No difference was observed in any point of interest in the level of TBARS, neither in the control group (16.19 ± 2.28 at S versus 14.72 ± 2.06 at R1 versus 13.47 ± 1.76 at R2) nor in the KCN group (16.59 ± 2.28 at S versus 15.6 ± 2.29 at R1 versus 14.32 ± 1.91 at R2) (Figure 2(a)).

#### 3.2.2. Levels of Nitrites (NO_2_^−^)

In the control group, there was a significant increase in the level of nitrites at R2 (66.3 ± 7.98) compared to R1 (56.36 ± 6.16) and S (53.14 ± 5.66). While in the group treated with KCN, we observed a significant increase at R1 (60.07 ± 7.03) compared to S (51.14 ± 5.66), while R2 (53.07 ± 1.2) was lower when compared to R1. At R2, a significantly lower level of NO_2_^−^ in the KCN group (53.07 ± 1.2) was noticed compared to that in the control group (66.3 ± 7.98) (Figure 2(b)).

#### 3.2.3. Levels of Superoxide Anion Radical (O_2_^−^)

An increase in O_2_^−^ production at R1 (92.91 ± 11.25) and R2 (89.63 ± 10.83) compared to S (74.17 ± 10.69) was found in the control group. Furthermore, in the KCN group, there was a decrease in the level of O_2_^−^ at R2 (65.84 ± 7.94) compared to S (76 ± 10.69). KCN induced further a significant decline in the level of examined parameter at R2 (65.84 ± 7.94) compared to the control group (89.63 ± 10.83) (Figure 2(c)).

#### 3.2.4. Levels of Hydrogen Peroxide (H_2_O_2_)

Hydrogen peroxide did not vary significantly within points of interest in the control group (42.39 ± 7.06 at S versus 44.88 ± 3.43 at R1 versus 40.3 ± 5.46 at R2). On the other hand, KCN induced a significant drop in H_2_O_2_ at R2 (30.82 ± 4.29) compared to S (40.39 ± 7.06) and a rise at R1 (42.36 ± 3.72) in comparison to R2. At R2, significantly lower level of H_2_O_2_ was observed in the KCN group (30.82 ± 4.29) compared to the control group (40.3 ± 5.46) (Figure 2(d)).

#### 3.2.5. Catalase Activity (CAT)

**Figure 3:**
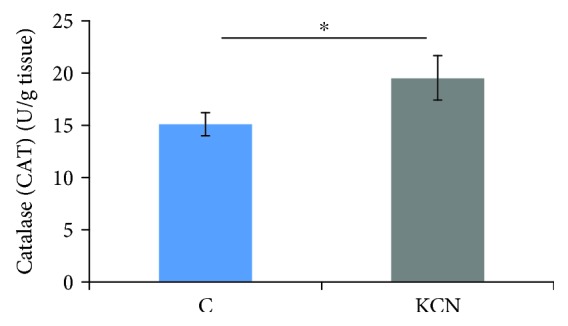
Effects of KCN postconditioning on catalase activity in heart tissue. Comparison between the control and KCN groups. ^∗^Statistical significance at the level of *p* < 0.05 between the control group and the KCN group; data are presented as means ± SE.

Application of KCN induced a significant increase in the activity of catalase (19.5 ± 2.1) when compared to the control group (15.1 ± 1.1) (Figure 3).

#### 3.2.6. Gene Expression in Heart Tissue

**Figure 4:**
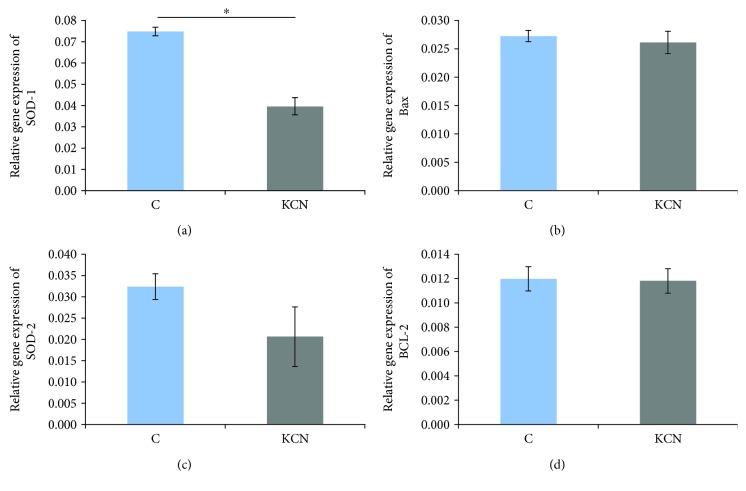
Effects of KCN postconditioning on gene expression in heart tissue. (a) Relative expression of SOD-1 gene in the control and KCN groups, (b) relative expression of Bax gene in the control and KCN groups, (c) relative expression of SOD-2 gene in the control and KCN groups, and (d) relative expression of Bcl-2 gene in the control and KCN groups. ^∗^Statistical significance at the level of *p* < 0.05 between the control group and the KCN group; data are presented as means ± SE.

Perfusion of the hearts with KCN led to a significant decrease in expression of gene SOD-1 (0.039 ± 0.02) in comparison to the control group (0.074 ± 0.01) (Figure 4(a)). However, KCN did not induce a significant change in gene expression of Bax (0.027 ± 0.01 in the control group versus 0.026 ± 0.003 in the KCN group) (Figure 4(b)), SOD-2 (0.031 ± 0.002 in the control group versus 0.021 ± 0.004 in the KCN group) (Figure 4(c)), and Bcl-2 (0.012 ± 0.006 in the control group versus 0.012 ± 0.005 in the KCN group) (Figure 4(d)), compared to the control group.

#### 3.2.7. Cytochrome c Determination

**Figure 5:**
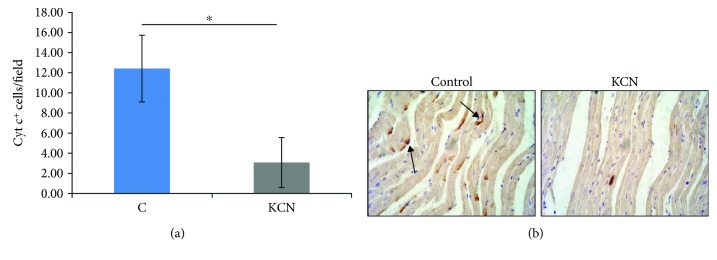
Effects of KCN postconditioning on cytochrome c expression in heart tissue. (a) Comparison between the control and KCN groups. ^∗^Statistical significance at the level of *p* < 0.05 between the control group and the KCN group; data are presented as means ± SE. (b) Cytochrome c immunohistochemistry of heart tissues from the control and KCN groups.

The mean count of positive stained cells reduced for 73.3% in the KCN group (3.08 ± 2.5) in comparison to the control group (12.42 ± 3.32) (Figure 5).

## 4. Discussion

Pharmacological PostC is proposed as great and practicable strategy to protect the ischemic heart with the similar protective effect as preconditioning [20]. Due to the unpredictability of clinical acute myocardial infarction, PostC may have greater clinical potential than preconditioning [8]. It has been reported that prolonged ischemia is accompanied by impairment in mitochondrial function, such as a decline in CcO activity [21, 22]. Generation of ROS in ischemic conditions affects the activity of enzymes composing the respiratory chain complexes, thus contributing to cardiac damage [23]. Nevertheless, phosphorylation of CcO and inhibition of its activity, as well as suppression of aerobic respiration and ATP utilization, may be involved in prevention of I/R-induced myocardial dysfunction [12]. In that sense, we hypothesized that KCN, as a selective inhibitor of CcO, may ameliorate the function of the heart which was exposed to I/R, when applied at the very onset of reperfusion. To the best of our knowledge, so far, there are no data regarding the effects of KCN in a pharmacological model of postconditioning and mechanisms responsible for potential benefits in cardioprotection.

Our results clearly show that in the first minute of reperfusion, contractility force and heart rate were increased in control condition, but still, these parameters started to decrease over 5 minutes of reperfusion. A drop in dp/dt max and coronary flow during recovery period indicates that vasculature dilated in accordance with the demands of heart contraction. Impaired coronary flow and heart rate in the control group at the end of reperfusion confirmed that ischemia impairs myocardial function. On the other hand, application of KCN, in the first minute of reperfusion, gradually restored cardiac contractility and systolic function, and as reperfusion continued, they progressively improved, manifested as an increase in dp/dt max and SLVP at the end of the experiment. Additionally, dp/dt min reached more negative values after accomplishing 5 minutes of reperfusion, thus suggesting that KCN also enhanced the lusitropic property of the heart. This agent prevented ischemia-induced decrease in heart rate and coronary flow. In fact, unchanged heart rate enabled sufficient time for the heart to effectively contract.

Most of the studies which examined the effects of KCN on cardiac function were mainly conducted on a model of preconditioning, while literature data referring to its effects in early reperfusion period is lacking. It was previously shown that 0.5 mmol/L KCN in cardioplegic solution enhanced the cardiac function by increasing the pressure and contractility and did not affect coronary flow, which correlates with our results [24]. These authors assumed that increased left ventricular contractility was the consequence of the myocardial norepinephrine release as a stress response and increase of intracellular calcium as well. Other authors also confirmed the cardioprotective effects of KCN, however, in a model of preconditioning [12].

The special focus of our investigation was the role of oxidative stress in the protective effects of KCN in a model of PostC, since overproduction of prooxidants has been reported as a major mechanism underlying I/R injury in the heart [25]. Enhanced production of prooxidants, such as O_2_^−^ and NO_2_^−^, was noticed in the control group during reperfusion, which was expected since restoration of oxygenated blood flow to the ischemic heart is paradoxically followed by increased release of ROS and oxidative stress [26]. In addition, I/R with or without KCN perfusion did not affect lipid peroxidation, while generation of H_2_O_2_ and O_2_^−^ decreased at the end of reperfusion in the KCN-treated group. NO, included in regulation of coronary flow, probably interacted with excessively produced O_2_^−^, which may explain unchanged values of O_2_^−^ noticed at the beginning of restoration of flow in the KCN group.

O_2_^−^/H_2_O_2_ dynamic can not be interpreted independently of the activities of enzymes of antioxidant defense, such as CAT and SOD. We observed lower expression of SOD-1 gene (CuZnSOD), found to be present in the cytosol, nucleus, and the intermembrane space of mitochondria, in the group treated with KCN [27]. However, acute application of KCN during reperfusion did not significantly affect the expression of SOD-2 (MnSOD) gene located in mitochondria. O_2_^−^ formed in mitochondria may be converted to H_2_O_2_ either in a spontaneous manner or in a reaction catalyzed by SODs [28, 29]. Another source of O_2_^−^ and H_2_O_2_ is conversion of xanthine dehydrogenase to xanthine oxidase. CAT catalyzes the decomposition of H_2_O_2_ to water and oxygen, and its higher activity in the KCN group supports a decrease in O_2_^−^ level [28]. Findings of the previously mentioned research suggest that KCN pretreatment induced a dose-dependent decrease in generation of free radicals and improvement in antioxidant capacity [12].

The main source of ROS production is certainly mitochondria, where CcO during ischemic injury contributes 30–35% of total prooxidant formation [20, 29]. This enzyme is responsible for transferring electrons from cytochrome c to O_2_^−^, and under physiological conditions, activity of CcO reflects an oxidative capacity of the cells [11, 30]. We measured expression of cytochrome c in heart tissue and showed a reduction of 75% in the KCN group (Figure 5). I/R damage, mediated by CcO hyperactivity and increased ROS production, is also followed by release of cytochrome c from mitochondria to cytosol, which initiates apoptosis and cell death [31, 32]. So, decrease of cytochrome c expression reflects decline in CcO activity, which correlates with our results. Since increased production of O_2_^−^ leads to enhanced activity of SOD, a drop in O_2_^−^ may explain the absence of change or a decrease in gene expression for SODs in the KCN group [33]. Additionally, it has been reported that CcO reduces NO_2_ to NO under hypoxic conditions, and inhibition of CcO may be a reason for high levels of nitrites detected during the first minute of reperfusion after KCN perfusion [34]. Taking into consideration that modulation of CcO activity may alleviate myocardial injury and increased oxidative stress, we may assume that observed effects of KCN in our investigations are attributed to lowering cytochrome c expression [21].

Five minutes of ischemia is not sufficient to induce myocardial necrosis, and in fact, all observed positive effects of KCN in our investigation are not attributed to a reduction of infarct size [13]. Reperfusion of ischemic heart stimulates opening of the mitochondrial permeability transition pores and leads to a mitochondrial release of cytochrome c, which is considered as one of the imperative events involved in apoptosis in these conditions [35]. Cardiomyocyte apoptosis in I/R leads to a further impairment of myocardial function and agents able to suppress it may significantly contribute to minimization of cardiac injury [36]. We investigated expression of apoptosis suppressor gene Bcl-2 and proapoptotic Bax gene after KCN application; however, this agent did not significantly affect gene expression [37].

Besides the fact that the protective effects of pharmacological PostC on myocardial function have been proven so far, it should not be forgotten that comorbidities and chronic therapy in humans may affect the signaling pathways included in preserving myocardial function [38]. This study may be a starting point for further researches which would fully clarify the effects of KCN on cardiac function in different models of PostC.

## 5. Conclusion

Based on our results, we may conclude that KCN led to the improvement of functional recovery of cardiac contractility and systolic and diastolic function when applied during the period of reperfusion. Additionally, KCN application was associated with the less production of prooxidants, which indicates alleviation of oxidative stress as one of the possible mechanisms through which KCN triggers cardioprotection during I/R injury. It is certainly necessary to further examine all possible mechanisms and potential adverse effects, but for now, KCN has shown promising effects on acute myocardial infarction as a pharmacological PostC agent.

## Figures and Tables

**Table 1:** Primers used for qRT-PCR analysis.

*β-Actin*	gatcagcaagcaggagtacgat	gtaacagtccgcctagaagcat
*Bax*	gctacagggtttcatccaggat	atgttgttgtccagttcatcgc
*Bcl-2*	gcaaagcacatccaataaaagcg	gtacttcatcacgatctcccgg
*SOD-1*	tgaagagaggcatgttggagac	cacacgatcttcaatggacaca
*SOD-2*	aatcaacagacccaagctaggc	cacaatgtcactcctctccgaa

## Data Availability

The data used to support the findings of this study are available from the corresponding author upon request.
